# The Negative and Positive Aspects of Employees’ Innovative Behavior: Role of Goals of Employees and Supervisors

**DOI:** 10.3389/fpsyg.2018.01871

**Published:** 2018-10-01

**Authors:** Ying Zhang, Jian Zhang, Jacques Forest, Chunxiao Chen

**Affiliations:** ^1^Donlinks School of Economics and Management, University of Science and Technology Beijing, Beijing, China; ^2^Department of Organization and Human Resource Management, Université du Québec à Montréal, Montreal, QC, Canada

**Keywords:** innovative behaviors, relationship conflict, in-role job performance, extrinsic goals, performance goals

## Abstract

We aim to examine the negative (relationship conflict) and positive (in-role job performance) outcomes of employees’ innovative behavior and explore the moderation effect of employees’ goal content and supervisors’ achievement goal orientation in these relationships. Data from 218 employees and their immediate supervisors were collected in companies in China and results show that employees’ innovative behaviors are positively related to their relationship conflict and in-role job performance, and employees’ extrinsic goals and supervisors’ performance goal moderate these relationships. Specifically, employees’ innovative behaviors were significantly and positively related to relationship conflict when either employees have high extrinsic goals or supervisor have high performance goals or both; and when supervisor have low level of performance goals, employees’ innovative behaviors were significantly and positively related to their in-role job performance. We contribute in showing when there are positive and negative outcomes of employees’ innovative behaviors and document the effect of moderating factors that may strengthen these benefits and lower the conflicts.

## Introduction

Employees’ innovative behaviors are defined as the generation, promotion, and realization of new ideas in products and processes, which is different from the concept of creativity which only focuses on the generation of new and useful ideas (Janssen et al., 2004; Shin et al., 2017). The general presumption is that employees’ innovative behaviors are always beneficial in order to do things better and are considered as an important source of an organization’s competitive advantage (e.g., Anderson et al., 2014; Shin et al., 2017). This is why most studies have focused on identifying factors that promote innovative behaviors (e.g., Zhou and Shalley, 2003; Wu et al., 2014; Wang et al., 2015), and some other studies have begun exploring the effects of innovation as a valuable independent variable (e.g., Janssen, 2003; Harrison and Wagner, 2016).

Janssen et al. (2004) gave an overall theoretical model of the bright sides (e.g., constructive conflict, performance improvement, positive job attitude, and well-being) and dark sides (e.g., destructive conflict, lowered performance, negative job attitude, and stress) of individual innovation, and suggested that researchers need to develop models to explore both the positive and negative outcomes of employee innovation. More and more studies now focus on the benefits and/or the interpersonal price that individuals and organizations may gain or pay for taking innovative behaviors (Janssen, 2003; Janssen et al., 2004; Gino and Ariely, 2012). For example, Gino and Ariely (2012) found that a creative personality and a creative mindset could lead to unethical behaviors by promoting an individuals’ ability to justify their behaviors. Harrison and Wagner (2016) suggest that creative behaviors at work predict less time spent with a spouse at home which affects the relationship. Moreover, a meta-analysis from Harari et al. (2016) showed that innovative behaviors were positively related to task performance and organizational citizenship behaviors, and negatively related to counterproductive work behaviors. Those theoretical and empirical evidences show that innovative behaviors are not always beneficial; they also have some hidden costs or damages. As mentioned by Anderson and Gasteiger (2008), p. 422, innovation have a dysfunctional aspect, which is always less visible, but has surfaced repeatedly across empirical studies. If it is not correctly managed, then it can potentially be seriously harmful to individuals, work teams, and even to the organization. Based on Janssen et al. (2004) model, the first aim of the current study is to explore the effect of innovative behaviors on employees’ relationship conflict, which could have influential effect on employees themselves and on their in-role performance as evaluated by their supervisors. This is important for the organizations in order to know how to efficiently manage employees’ innovative behaviors.

Another important aim of our study is to explore the reasons why employees sometimes gain profits, and other times pay the price, for engaging in innovative activities. Janssen et al. (2004) suggest that some moderators could activate the benefits and hinder the costs of the outcomes of employee innovation. For example, they suggested that innovators’ characteristics such as traits, states, goals, needs, abilities, and skills might moderate innovative efforts. In this line of thoughts, we argue that employees’ goal content (Deci and Ryan, 2000) could be an important innovators’ characteristic that could regulates the outcomes of employee innovation. This is because employees’ goal content is their work-related reinforcement preferences which could determine individuals’ behavioral choices in the workplace (Malka and Chatman, 2003). Based on self-determination theory (SDT), Kasser and Ryan (1993, 1996) distinguished goals between intrinsic (such as community contribution and health) and extrinsic (such as fame and financial success) goals. Numerous studies suggest that employees different goal content is an important individual factor which could influence job outcomes, including conflicts and in-role job performance (e.g., Vansteenkiste et al., 2006; Zhang et al., 2016). Thus, we aim to explore if employees’ different goal pursuits could strengthen and/or weaken the relationship between employee’s innovative behaviors and positive and negative job outcomes.

When exploring the outcomes of innovative behaviors, supervisors’ effect should not be neglected because they often play a key role in the process of employees’ innovative behaviors (Sijbom et al., 2015a,b). Studies begin to explore how supervisors facilitate employees to perform innovatively or hinder them from doing so and suggest that supervisors’ reactions to subordinates’ innovative behaviors would be bound with supervisors’ own achievement goal pursuits (Yukl, 1989; Sijbom et al., 2015b). Achievement goal pursuits are believed to create different perceptual-cognitive frameworks for how people define, interpret, and respond to achievement situations (Van Yperen and Orehek, 2013), and these goals have been regrouped in two main orientations: a mastery orientation (mastering a task or skill) and a performance orientation (being the best at a task or skill). Supervisors’ achievement goals tend to affect supervisors in the way they interpret, experience, and respond to employees’ innovative input (Sedikides and Strube, 1997; Sijbom et al., 2015b). Moreover, scholars have showed that supervisors with performance goals, relative to supervisors who have mastery goals, were more likely to oppose subordinates’ radical creative ideas (Sijbom et al., 2015b). Based on this, we investigate how supervisors’ achievement goals influence the relationships between employees’ innovative behaviors and outcomes.

Thus, the contributions of this study are twofold. First, we seek to test the outcomes of employees’ innovative behaviors and explore if individual innovation could explain the benefits (e.g., improving in-role job performance) or costs (increase in relationship conflicts) in the workplace, and adds to the current innovation theories by investigating the outcomes of innovative behaviors from both a positive and negative point of view. Second, we aim to explore how some moderating factors may strengthen these benefits and diminish the costs, and add to the management practice by suggesting how individuals and organizations can gain the profits and avoid the costs in stimulating innovative behaviors in the workplace.

## Hypotheses Development

### Individual Innovative Behavior and Relationship Conflict

The focus of employees’ innovative behaviors includes both the generation and the implementation of the new ideas, which encompasses a wider range of actions than creativity (Shalley et al., 2004). The scope of innovation is quite broad, as it ranges from small changes that just modify or improve the daily work processes to the influential new ideas and processes that could have effect on theories, practices, or products across the whole organization (Janssen, 2003). Past researchers focused on exploring the factors that could promote individual innovative behavior (Anderson et al., 2014; Wang et al., 2015). However, our study focuses on the outcomes of employees innovative behavior and identify if it is more likely that employees will pay the price (or gain from) performing innovative activities.

Relationship conflicts are occurring among employees who have interpersonal disagreements regarding some issues (Meier et al., 2013), such as, political preferences, values, and interpersonal styles just to name a few. Janssen (2003) makes an initial attempt to test the positive relationship between employee’s innovative behaviors and conflicts with co-workers. First, co-workers are oftentimes inclined in avoiding the insecurity and stress that changes brings. Second, co-workers that are used to their daily familiar practices and actions are reluctant to change them. Third, co-workers often have already invested their commitment in the usual way of doing things in the organization. Thus, co-workers’ resistance to the innovative behaviors will further lead to more relationship conflicts with the employees who are willing to put forward new ideas. Besides, Harrison and Wagner (2016) suggests that creative work might use up the resources that are essential for personal relationships. Employees who engage in creative activities need to fully concentrate on the problem at hand for long bouts of time, which leads them to not have much energy remaining in order to maintain a healthy relationship with their colleagues (Policastro and Gardner, 1998). Finally, the implementation of individual innovations may require employees to perform some new tasks and procedures that will increase their workload and result in them feeling tension and anxiety (González-Romá and Hernández, 2016). Thus, the following hypothesis is proposed:

Hypothesis 1: Employees’ innovative behaviors are positively related to relationship conflicts.

### Individual Innovative Behavior and In-Role Job Performance

In-role job performance can be defined as activities that are related to the employees’ formal role requirements or tasks that are specified in a job description (Borman and Motowidlo, 1997). Some studies argue that individuals’ innovative behaviors could enhance in-role job performance (e.g., Walberg and Stariha, 1992). Harari et al. (2016) conducted a meta-analysis and suggest that innovative behaviors were positively related to in-role performance (ρ = 0.55). Thus, we suggest that the relationship between innovative behaviors and in-role job performance is positive. Innovative behaviors are more or less always introduced to solve a workplace issue; it thus means that when employees face some problems in their work-related tasks, they are more likely to engage in the innovative behaviors because they believe that developing a new method may be helpful to fix the current problem (Yuan and Woodman, 2010). Thus, it is likely that innovative behaviors can facilitate in-role performance. Besides, employees’ innovative behaviors and in-role job performance belong to the same general dimension of job performance (Harari et al., 2016). Although innovative behaviors have been usually operationalized as a unique dimension in the literature, these two dimensions may have some overlaps. This is because the two dimensions share some of the same determinants; for example, cognitive ability and job knowledge could be predictors of both innovative behaviors and in-role performance (Salgado et al., 2003; Kuncel et al., 2004). Hence, the following hypothesis is proposed:

Hypothesis 2: Employees’ innovative behaviors are positively related to their in-role job performance.

### A Relatively More Extrinsic Goal Pursuit of Employees as a Moderator

Malka and Chatman (2003), p. 739, defined work goal orientations as “work-related reinforcement preferences, or tendencies to value specific types of incentives in the work environment” (see also Vansteenkiste et al., 2007). Kasser and Ryan (1993, 1996) distinguished goals that are intrinsic (such as community contribution, health, personal growth, and affiliation) from others which are extrinsic (such as fame, financial success, and physical appearance) (Deci and Ryan, 2000). Specifically, intrinsic goals reflect individuals’ natural growth tendencies and are characterized by an inwardly oriented mindset. By contrast, extrinsic goals have an “outward” orientation (Williams et al., 2000) – that is, they focus on obtaining contingent approval or the external manifestations of worth rather than the satisfaction of basic psychological needs. Previous studies found that a high valuation of extrinsic relative to intrinsic goals would yield many negative outcomes, such as poorer quality friendships and love relations (Kasser and Ryan, 2001) and less cooperation when resources are scarce (Sheldon et al., 2000).

Extrinsically oriented individuals objectify others (e.g., primarily consider colleagues as their competitor) and use them as instruments to attain their own material (or social) values rather than developing a close and trustful relationship with others, they are thus more likely to experience their friendships as more conflictual (Kasser and Ryan, 2001; Kasser, 2002; Vansteenkiste et al., 2006). Past studies show that extrinsic goals are associated with fewer prosocial actions (Sheldon and Kasser, 1995), greater disagreeableness (Roberts and Robins, 2000), as well as less cooperation (Sheldon et al., 2000), which are all conducive to objectification and interfere with the quality of interpersonal relationships (Kasser et al., 2007). When an employee with a strong focus on extrinsic relative to intrinsic goal pursuits (E/I) implement his new ideas, he tends to consider their colleagues as instruments and use them as efficiently as possible to attain his own ambitions. This will likely increase co-workers resistance to innovative behaviors and will further lead to more relationship conflicts. Besides, evidence suggests that innovative work might use up the resources that are usually essential for other activities (Harrison and Wagner, 2016) Extrinsically oriented individuals are thus more likely to compete (rather than cooperate) with colleagues when common resources are scarce (Sheldon et al., 2000), thus leading to more relationship conflicts. The following hypothesis is thus proposed:

Hypothesis 3: Employee’s E/I moderate the relationship between employees’ innovative behaviors and their relationship conflicts, such that this relationship will be more positive when employee have high level of extrinsic goal orientation, and less positive when employee have low level of extrinsic goal orientation.

Although researchers suggested that extrinsic goals were associated with more negative outcomes, pursuing extrinsic goals (money and fame) is ubiquitous among employees as work is often the sphere of life where employees get money to live (Zhang et al., 2016). In numerous organizations, in-role job performance is an important factor in determining employees’ pay, since it has a closer relationship to monetary concepts such as payment and salary because both the quality and quantity of tasks are linked to the individuals’ salaries (Zhang et al., 2015). Zhang et al. (2018) found that employees’ extrinsic, relative to intrinsic, work goal orientations could positively predict in-role job performance. This is because the benefits associated with attaining extrinsic goals are always relatively short-lived (Niemiec et al., 2009; Zhang et al., 2018), and employees are more likely to set new extrinsic goals quickly, leading extrinsic goal-oriented people to become trapped in a “hedonic treadmill” (Vansteenkiste et al., 2008). This treadmill continuously encourages individuals to increase their job performance to obtain extrinsic goals. When the employees tend to improve their in-role job performance to attain their extrinsic goals, they will put more effort in the innovation activities; thus, employees’ strong extrinsic goals pursuits will be more helpful in moderating the positive relationship between their innovative behaviors and in-role performance. Accieding, the following hypothesis is proposed:

Hypothesis 4: Employee’s E/I moderate the relationship between employees’ innovative behaviors and their in-role job performance, such that this relationship will be more positive when employee have high level of extrinsic goal orientation, and less positive when employee have low level of extrinsic goal orientation.

### Supervisors’ Performance Goal Orientation as a Moderator

Achievement goal research focus on exploring how individual define, experience, and respond to competence-relevant situations in individual task settings (Van Yperen and Orehek, 2013; Sijbom et al., 2016). Achievement goals are traditionally separated in two broad orientations: a mastery orientation and a performance orientation. A mastery orientation focuses on developing and gaining competence by acquiring new skills and doing one’s best, whereas performance goals reflect the desire to demonstrate superior competence by outperforming others (Van Yperen, 2003; Janssen and Van Yperen, 2004). Studies suggest that supervisors’ achievement goals could be an important factor that influence their opposition and/or adoption of subordinates’ creative ideas (Sijbom et al., 2015a,b). We also think that supervisors’ achievement goals could be a factor that moderate the relationships between innovative behaviors and outcomes.

Previous research showed that performance goal supervisors always focus on an interpersonal and comparative standard in defining competence and evaluating performance; they tend to perceive subordinates’ competence level as a potential menace to themselves (Sijbom et al., 2016). When subordinates suggest new ideas or implement innovation that challenges the *status quo* of routines, performance goal supervisors are more likely to perceive subordinates innovative behavior as a threat to their competence as a leader (Detert and Burris, 2007; Mast, 2010). Besides, performance goal of supervisors leads them to try to demonstrate their superior leadership performance relative to their subordinates and their colleagues. It may also push them to view the current thoughts and routines, which they established in their daily managerial work as supervisors, being a competence demonstration (Sijbom et al., 2015a). Thereby, subordinates who point out problems and tend to implement the new ideas for doing things may give the impression that their supervisors’ competencies are challenged and questioned (Sijbom et al., 2015b). Thus, supervisors pursuing performance goals may not tend to integrate, or might even oppose, to subordinates’ innovative ideas. Based on the previous reviews, co-workers are more likely to resist to their colleagues’ innovative behaviors and lead to more relationship conflicts with them; we propose that these relationship conflicts will be stronger when subordinates’ innovative behaviors even cannot get the support from their supervisors. Thus, the following hypothesis is proposed:

Hypothesis 5: Supervisors’ performance goal moderates the relationship between employees’ innovative behaviors and relationship conflicts, such that this relationship will be more positive when leader have high level of performance goal orientation.

And since both supervisors’ performance goal and employees E/I goal could positively moderate the relationship between employees’ innovative behaviors and their relationship conflicts, we also propose a three-way interaction effect:

Hypothesis 6: The relationship between employees’ innovative behaviors and relationship conflicts will be more positive when both the supervisors have high level of performance goal and employees have high level of extrinsic goal orientation.

Resource allocation theory suggests that individuals have finite resources (Hobfoll, 2002), and engaging in an innovative process requires a lot of resources’ consumption which will come at the expense of other activities. As a result, employees are not able to apply equivalent resources toward both innovative activities and in-role job performance, where an emphasis placed on the former could detract from the later (Scott, 1995; Harari et al., 2016). Thus, a competing perspective suggests that although innovative behavior is aimed at improving performance, the innovative activities may consume and use the attention and resources that are necessary to in-role job performance, which lead to a smaller relationship between innovative behavior and in-role job performance (Harari et al., 2016). We proposed that supervisors can influence this relationship. That is, supervisors usually control the resources and have key power to make the decisions to allocate the resources (Dewar and Dutton, 1986). However, supervisors pursuing performance goals may not support subordinates’ innovative behaviors, and they may not allocate resources to subordinates’ innovative activities. These subordinates then allocate their energy and resources to innovative activities, but deplete their stock of resources available for in-role job performance. Thus, the following hypothesis is proposed:

Hypothesis 7: Supervisors’ performance goal moderate the relationship between employees’ innovative behaviors and in-role job performance, such that this relationship will be weaker when leader have high level of performance goal orientation.

## Ethics Statement

The study was reviewed and approved by the ethical committee of University of Science and Technology Beijing, Donlinks School of Economics and Management. Written informed consent was obtained from all employees and their managers. All participants were informed of their right to withdraw from the survey at any time.

## Materials and Methods

### Participants and Procedure

A total of 295 employees from diversified companies in China were surveyed. Every organization’s HR manager helped us to collect the data by preparing a list of selected employees and their supervisors. Employees filled in the questionnaires to measure their individual innovation, their relationship conflict and their personal goal content orientations. The corresponding managers completed the questionnaires to assess their own achievement goal orientation and their subordinates’ innovation and in-role job performance. There were 218 effective matching responses (response rate of 74%).

### Measures

Unless otherwise noted, responses to all items were measured on seven-point Likert-type scales, ranging from strongly disagree (1) to strongly agree (7).

#### Innovative Behavior

Employees’ innovative behaviors were assessed using Janssen’s (2003) nine-item scale in the workplace. This scale includes three sub-scales with three items each, (1) idea generation, (2) idea promotion, and (3) idea realization, which draws on Kanter’s (2000) work on the stages of innovation. In our study, we asked both employees and their immediate supervisors to rate the nine innovative work behaviors in the workplace. Sample items are “Searching out new working methods, techniques, or instruments (idea generation),” “Mobilizing support for innovative ideas (idea promotion),” and “Introducing innovative ideas into the work environment in a systematic way (idea realization).” We use the combination of the three subscales to create an overall score of innovative work behaviors. The Cronbach’s alpha for the self-rated and supervisor-rated scale were 0.95 and 0.94, respectively.

We measured employees’ innovative behaviors by both self-ratings and supervisors’ ratings, and we used the self-ratings score in our study for two reasons. First, to avoid for common method bias and the halo effect between supervisors’ evaluation of subordinates’ job performance and innovation (Harari et al., 2016). Since employees’ in-role job performance and innovation were both rated by supervisor, there is a strong correlation between the innovation and in-role performance evaluation (*r* = 0.49, *p* < 0.01). Second, supervisors may judge employees’ innovative ideas to be unrealistic or as undesirable suggestions and hence view their creativity less positively (Janssen and van der Vegt, 2011). In addition, our study aims to explore the outcome of innovative behavior. It is thus more reasonable to use self-ratings of innovation in our particular case.

#### Employees’ Goal Content

We used a Chinese version (Tang et al., 2008) of Kasser and Ryan’s (1996) Aspiration Index that assess the importance of the extrinsic goals of financial success, and social recognition, and the intrinsic goals of health, self-acceptance, affiliation, and community feelings. Each goal was measured with five items. The original Aspiration Index is a 35-item measure and in this study, we adapted it to the work context. For this, we had to remove the “appealing appearance” (Kasser and Ryan, 1996) which is much more difficult (if not impossible) to transpose in the work context. A sample item for extrinsic goal includes “I want to be financially successful (financial success)” and a sample item for intrinsic goal includes “work to make the organization a better place (community feelings).” The Cronbach’s alpha for extrinsic and intrinsic goal were 0.86 and 0.94, respectively.

The intrinsic and extrinsic goal subscales were significantly positively correlated (*r* = 0.57, *p* < 0.01), suggesting that employees who attach high importance to extrinsic goals also tend to attach importance to intrinsic goals. To compare peoples’ goal systems as a whole, studies commonly compute a “relative extrinsic versus intrinsic value orientation” score by subtracting intrinsic from extrinsic goal scores or add an overall work goal scale as a control variable (Duriez et al., 2007; Vansteenkiste et al., 2007). This approach is used because extrinsic goals are not considered negative in themselves, but are thought to become problematic when they become more prominent than intrinsic goals in a person’s overall goal orientation (Sheldon and Krieger, 2014). Thus, we calculated an overall work goal scale by summing the 30 items and put it as a control variable.

#### Supervisors’ Achievement Goals

We measured mastery and performance goal orientation items with 19 items (11 for mastery and eight for performance; Van Yperen and Janssen, 2002). An example of item for mastery goal is “I feel successful on my job when I feel I am improving,” and a sample item for performance goal is “I feel successful on my job when I perform better than my colleagues.” The Cronbach’s alpha for mastery orientation and performance orientation were 0.86 and 0.71, respectively.

Past studies suggest that individuals may pursue achievement goals simultaneously or subsequently, and in a specific context, one particular achievement goal may be an individual’s dominant achievement goal (Van Yperen et al., 2011; Van Yperen and Orehek, 2013). We found in our study that the mastery orientation and performance orientation subscales were significantly positively correlated (*r* = 0.49, *p* < 0.01), suggesting that supervisors who have high orientation on performance goals also tend to have high orientation on mastery goals. Thus, we calculated an overall achievement goal scale by summing the 19 items and put it as a control variable.

#### Relationship Conflicts

We used four items to assess employees’ relationship conflicts with their colleagues that were developed by Janssen (2003). A sample item is “Do you and your co-workers have different ideas on job matters?”. The Cronbach’s alpha for the scale is 0.82.

#### In-Role Job Performance

We use Podsakoff and MacKenzie’s (1989) five-item scale to measure employees’ in-role job performance. The immediate supervisors of the employees indicated the extent of the quality and quantity of the employees’ in-role activities. A sample item is “This worker fulfills all responsibilities required by his/her job.” The Cronbach’s alpha for the scale is 0.70.

#### Covariates

To control for the possibility that other variables may influence the relationships between the predictor and outcome variables (Janssen, 2003; Sijbom et al., 2016), we controlled for several variables, including age, gender (0 = male and 1 = female), education (1 = high school, 2 = college, and 3 = masters and doctoral degree), and position level (1 = entry level employee, 2 = middle level employee, and 3 = senior supervisors).

## Results, Analysis, and Discussion

### Results

We applied a confirmatory factor analysis to demonstrate the unique factor structure of each study measure. **Table 1** shows that the indexes of the five factors model are better than the alternative models, thus confirming the uniqueness of each independent variables. The means, standard deviations, and correlation coefficients of all the variables are in **Table 2**, where employees’ innovative behaviors are positively correlated to relationship conflicts (*r* = 0.46^∗∗^, *p* < 0.01) and their in-role job performance (*r* = 0.22^∗∗^, *p* < 0.01). Results also show that the correlation between supervisors’ performance goals and employees’ extrinsic goals was not significant.

**Table 1:** The result of confirmatory factor analysis of the model.

Models	χ^2^/df	RMSEA	CFI	IFI	NFI
Five factors model	1.86	0.06	0.92	0.93	0.85
Four factors model (1)	2.38	0.08	0.87	0.88	0.80
Four factors model (2)	2.34	0.79	0.87	0.78	0.81
Three factors model	2.96	0.95	0.82	0.82	0.76
Two factors model	4.04	0.12	0.72	0.72	0.66
One factor model	5.24	0.14	0.60	0.60	0.55

*Five factors: EI, SPG, EEG, RC, and IRP; four factors (1): EI, SPG, EEG, and RC + IRP; four factors (2): EI, SPG + EEG, RC, and IRP; three factors: EI, SPG + EEG, and RC + IRP; two factors: EI + SPG + EEG and RC + IRP; one factor: EI + SPG + EEG + RC + IRP; EI, employee’s innovation; SPG, supervisors’ performance goals; EEG, employees’ extrinsic goals; RC, relationship conflicts; IRP, in-role performance*.

**Table 2:** Correlations between variables.

Variables	*M*	*SD*	1	2	3	4	5
1. Employees’ innovation	4.73	1.01	(0.95)				
2.Supervisors’ performance goals	4.30	0.61	0.15^∗^	(0.71)			
3. Employees’ extrinsic goals	4.29	1.13	0.33^∗∗^	0.10	(0.86)		
4. Relationship conflicts	4.13	1.15	0.46^∗∗^	0.17^∗^	0.49^∗∗^	(0.82)	
5. In-role performance	4.80	0.98	0.22^∗∗^	0.11	-0.04	-0.05	(0.70)

*^∗^*p* < 0.05, ^∗∗^*p* < 0.01*.

We used a linear mixed model to test Hypotheses 1 and 2. As **Table 3** (model 2) shows, over and above the effect of control variables, employees’ innovative behaviors were found to be positively and significantly related to their relationship conflicts (β = 0.43^∗∗^, *p* < 0.01) which lend support to Hypothesis 1. As **Table 5** (model 2) shows, over and above the effect of control variables, employees’ innovative behaviors were found to be positively and significantly related to their in-role job performance (β = 0.20^∗∗^, *p* < 0.01), which provides support for Hypothesis 2.

**Table 3:** Results of multilevel analyses testing hypotheses.

Predictors	Relationship conflicts			
	1	2	3	4
Gender	0.33*	0.19	0.15	0.14
Age	0.01	0.01	0.01	0.01
Education	-0.27	-0.18	-0.17	-0.17
Position	0.03	-0.01	-0.04	-0.02
Overall employee goal orientation	0.49**	-0.48**	-0.36*	-0.37*
Overall leader goal orientation	0.12	0.01	-0.02	-0.01
Employees’ innovation		0.43**	0.45**	0.48**
Employees’ extrinsic goals		0.79**	0.71**	0.73**
Supervisors’ performance goals		0.10	0.08	0.12
EI*EEG			0.15**	0.14*
EI*SPG			0.18*	0.14*
EEG*SPG			-0.02	0.03
EI*EEG*SPG				-0.10*
∆*R*^2^		0.158**	0.056**	0.012*

*EI, employee’s innovation; EEG, employees’ extrinsic goals; SPG, supervisors’ performance goals; ^∗^*p* < 0.05,^∗∗^*p* < 0.01*.

**FIGURE 1:**
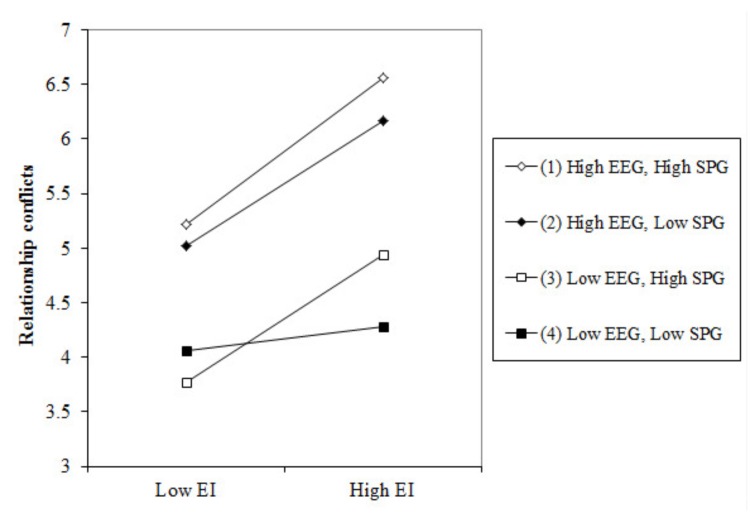
Three-way interaction effects of El by SPG and EEG on employee relationship conflicts.

To test Hypotheses 3, 5, and 6, we included overall employee goal content orientation, overall supervisors’ achievement goal orientation, the control variables, the main two-way interaction, and the three-way interaction effects (as fixed effects) in a linear mixed model to predict relationship conflicts. **Table 3** (model 4) shows that the two-way interaction between innovative behaviors and employees’ extrinsic goals, the two-way interaction between innovation and supervisors’ performance goals, and the three-way interaction all reached a significant level. To further analyze this interaction effect, we used the procedure outlined by Aiken et al. (1991) and run a simple regressions test. As **Table 4** and **Figure 1** show, employees’ innovative behaviors were significantly and positively related to relationship conflicts when either employees have high extrinsic goals or supervisors have high performance goals or both; the highest level of relationship conflicts was observed when employees extrinsic goals (EEG) and supervisors performance goals (SPG) were all high. Thus, Hypotheses 3, 5, and 6 were supported.

**Table 4:** Conditional relationship between EI and relationship conflicts at low and high values of EEG and SPG.

Employees’ innovation	Supervisors’ performance goals	Relationship conflicts	
		*B*	*T*
Low	Low	0.11	1.09
High	Low	0.58	3.93^∗∗^
Low	High	0.58	4.51^∗∗^
High	High	0.67	5.72^∗∗^

*^∗^*p* < 0.05, ^∗∗^*p* < 0.01*.

**FIGURE 2:**
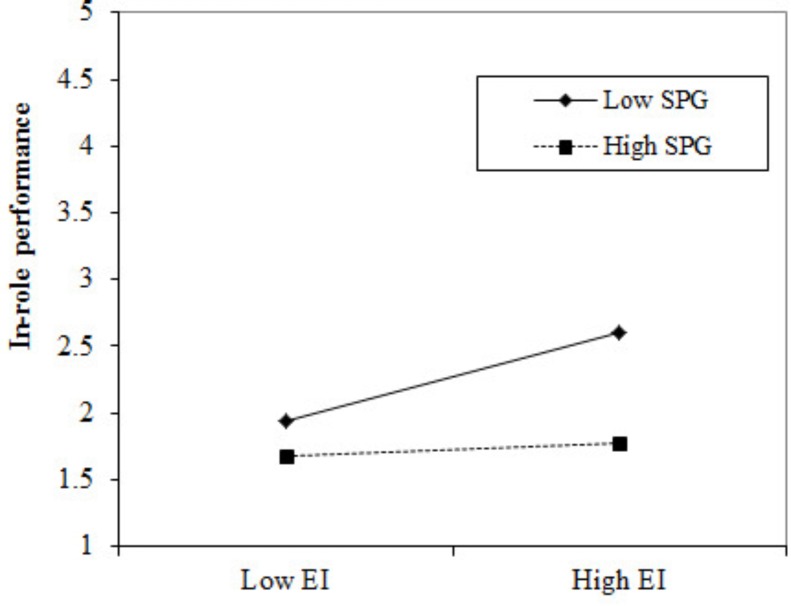
Two-way interaction effects of El by SPG on employee in-role performance.

We used the same steps to test Hypotheses 4 and 7 where we did not include the three-way interaction effects in the model because we hypothesized that high level of EEG strengthen, while high level of SPG weakens, the relationship between employees’ innovative behaviors and in-role job performance. **Table 5** (model 3) shows that only the two-way interaction between innovative behaviors and supervisors’ performance goals reached a significant level. **Figure 2** shows that when supervisors have low level of performance goals, employees’ innovative behaviors are significantly and positively related to their in-role job performance. Thus, Hypothesis 7 was supported.

**Table 5:** Results of multilevel analyses testing hypotheses.

Predictors	In-role performance		
	1	2	3
Gender	-0.125	-0.05	-0.04
Age	-0.01	-0.01	0.01
Education	0.37**	0.41**	0.39**
Position	-0.17	-0.13	-0.11
Overall employee goal orientation	0.04*	0.26	0.19
Overall leader goal orientation	0.43**	0.71**	0.73**
Employees’ innovation		0.20**	0.19**
Employees’ extrinsic goal		-0.31*	-0.26
Supervisors’ performance goals		-0.28**	-0.27*
EI*EEG			-0.05
EI*SPG			-0.14*
Δ*R*^2^		0.086*	0.029*

*EI, employee’s innovation; EEG, employees’ extrinsic goal; SPG, supervisors’ performance goals; RC, relationship conflicts; IRP, in-role performance; ^∗^*p* < 0.05, ^∗∗^*p* < 0.01*.

The purpose of this study was to explore the outcomes of employees’ innovative behaviors and how supervisors and employees themselves could influence the benefits or price of individual innovation. Using the data collected from supervisor-subordinate dyads, we found that employees’ innovative behaviors are positively correlated to their relationship conflicts and in-role job performance. The results of the present research underline the importance of not only focusing on the antecedents of innovative behaviors but also on the profits or price that individuals and organizations may gain or pay for initiating and implementing innovative activities. We further showed that employees’ innovations were more significantly and positively related to relationship conflicts when either employees have high extrinsic goals or supervisors have high performance goals or both are high of this variable. We also found that supervisors’ high level of performance goals weakens the relationship between employees’ innovation and their in-role job performance.

Our study contributes to the research literature on employee innovative behaviors in several ways. First, previous research focused on the factors for employee innovative behaviors as the benefits of it seem obviously compelling (Zhou and Hoever, 2014; Harrison and Wagner, 2016). Our research adds to the current studies that investigated the outcomes of innovative behaviors both from a positive and negative point of view. By doing so, we can provide a theoretical logic and empirical evidence showing that innovative behaviors could indeed leads to some positive outcomes, such as employees in-role job performance, but it could also produce negative effects by leading employees to have more relationship conflicts with colleagues. Importantly, our research provides a support for the positive relationship between innovative behaviors and in-role job performance. Past research have had competing perspectives suggesting that employees might have fewer resources available to in-role job performance after engaging in the innovative processes, which will lead to a smaller or null relationship between innovative behaviors and in-role job performance (Scott, 1995). Our results suggest that engaging in innovation by developing and implementing new ideas or by modifying existing procedures could facilitate in-role job performance. Our study also contribute in showing the usefulness of using multisource rating data (i.e., supervisors rated in-role job performance of employees, and employees rated their innovative behaviors in order to avoid the halo effect; Scullen et al., 2000).

Second, the present research contributes by providing evidence that innovator characteristics, such as their extrinsic goal pursuits, could moderate the relationship between employees’ innovative behaviors and relationship conflict. That is, a relatively high extrinsic goals pursuit of employees will produce and generate greater relationship conflicts when engaging in innovative activities. Previous studies just found that employees’ extrinsic and intrinsic goals could influence their job outcomes, such as job satisfaction and turnover intentions (Vansteenkiste et al., 2007; Unanue et al., 2017). Our study suggest that employees’ goal pursuit is also an important factor that can moderate the relationships between innovative behaviors and its outcomes.

Finally, the present findings also provide insights into the meaning of supervisors’ achievement goals for the management of employees’ innovative behaviors. Previous studies provided some evidence about the fact that supervisors’ achievement goals could influence their attitude (supportive or opposing) to subordinates’ creative inputs (Sijbom et al., 2015a,b). Our study has explored the influence of supervisors’ achievement goals on the process of innovation on outcomes and suggest that supervisors’ performance goal orientation could lead innovators to have more conflicts with colleagues and a weaker relationship with their in-role job performance. This finding highlights the effects of achievement goal on interpersonal variables and contribute to the current literature in showing that supervisors’ achievement goals can not only affect supervisors’ interpersonal behaviors but also affect subordinates’ behaviors and outcomes (Sijbom et al., 2015a,b, 2016).

### Limitations and Future Research

Beside its contributions, several limits need to be considered when interpreting the results of the present study, and future research suggestions put forward. First, Janssen et al. (2004) suggest that there are a broad variety of traits, states, values, needs, abilities, and skills of innovator characteristics that may moderate the benefits and costs of their innovative efforts. In our study, we only focused on individuals’ goal pursuit as a moderator. In fact, moderators could be a vital factor that helps explain the negative or positive outcomes of innovation, which means that organizations can focus on these moderators if they want to decrease the negative effect of innovation. Thus, in future studies, we need to explore others employees’ characteristic as potential moderators. Besides the employees and supervisors’ factors, we could also explore the moderating effect of organizational factors (Janssen et al., 2004) in future study. For example, a corporate culture that emphasizes cooperation versus competition could potentially have different moderating effect on innovation.

Second, we did not get the support for Hypothesis 4, which means that employees’ extrinsic goal pursuits do not strengthen the relationship between innovative behaviors and in-role job performance. This result is consistent with some other studies, which suggests that extrinsic goal pursuits will lead to more negative outcomes, such as higher emotional exhaustion and turnover intentions as well as lower well-being (Vansteenkiste et al., 2007; Dittmar et al., 2014). Future research may therefore investigate the moderating effect of both intrinsic and extrinsic goal pursuits, which could provide support for the effect of individual goal content. Finally, in this cross-sectional research, it was difficult to make any causal inferences; for example, some research suggests that conflicts could also have an influence on innovation and not the other way around (De Dreu, 2006). Thus, we suggest that, in future study, longitudinal and dynamic research need to be used in order to explore the directionality among these variables.

### Practical Implications

Innovative work behaviors are always seen as a critical determinant of organizational performance, success, and long-term survival (Anderson et al., 2014) and it is important and valuable for research on workplace innovation to offer insights into how it is possible to promote workplace creativity and achieve innovative outcomes. In this line of thoughts, the results from this study underlines some practical concerns. That is, managers and researchers should not only focus on encouraging the development of new and useful ideas or promotion of innovative actions but also pay attention to what decreases some of the costs of innovative work.

According to our result, innovative behaviors could be beneficial to employees’ in-role job performance, but they can also bring more relationship conflicts for them. We found that individual factors, namely employees’ extrinsic goals, and supervisors’ factor, namely, supervisors’ performance goals, could strengthen the conflicts that innovation stimulates. Thus, in order to avoid the setback of innovation, organizations should create an environment in which supervisors are encouraged to increase their mastery goals and decrease their performance goals. One way organizations can create a mastery goal motivational climate for supervisors is to put more emphasis on the progress and effort rather than on the outcomes (e.g., Van Yperen and Orehek, 2013; Sijbom et al., 2015b). Besides, our research also advances the idea that organization should promote intrinsic, rather than extrinsic, values among their employees. This idea might seem counterintuitive, as work is the main place where the majority of people get their money, but recent research put forward the ideas that it is not money itself, but more importantly the meaning money has (Landry et al., 2016) or the meaning that the rewards have (Landry et al., 2018), that has positive and/or negative effects. In this same stream of thoughts, some researchers suggest that an autonomy supportive environment may promote individuals’ intrinsic goal pursuit (e.g., Lekes et al., 2010), thus providing an autonomy supportive environment for employees could be a helpful mean to increase intrinsic goal pursuits among employees. Moreover when organizations recruit new employees, they can add some evaluation of the goal content with standardized questionnaire in order to select more intrinsically oriented employees.

## Conclusion

Most research on innovation only focus on examining the personal or contextual factors that enhance it because, without innovation, few organizations can survive, thrive, and prosper in the competitive environment we live in (Anderson et al., 2014). While a small number of studies evaluating the consequences of innovation is starting to emerge (Janssen, 2003; Harrison and Wagner, 2016), our results extend theory on innovation by exploring the outcomes that includes both the benefits and costs of it. Results also clarify under which conditions innovators sometimes gain profits, and at other times pay the price of pushing innovative ideas. This paper provides a foundation on which further research can help organizations understand how and when they can stimulate the positive effects, while simultaneously reduce the negative effects, of their employees’ innovative behaviors.

## Author Contributions

All authors (YZ, JZ, JF, and CC) substantially contributed to the research concept and design. YZ predominantly contributed to conduct the literature review, designed the studies, collected a part of data, analyzed the data, and drafted the manuscript. JZ and JF repeatedly revised and refined the content of the manuscript. CC contributed to help collect a part of the data and draft the manuscript.

## Conflict of Interest Statement

The authors declare that the research was conducted in the absence of any commercial or financial relationships that could be construed as a potential conflict of interest.
